# Processes, practices and influence: a mixed methods study of public health contributions to alcohol licensing in local government

**DOI:** 10.1186/s12889-018-6306-8

**Published:** 2018-12-18

**Authors:** Joanna Reynolds, Michael McGrath, Jessica Engen, Ghazaleh Pashmi, Matthew Andrews, Jin Lim, Karen Lock

**Affiliations:** 1 Faculty of Public Health & Policy, London School of Hygiene and Tropical Medicine, 15-17 Tavistock Place, London, WC1H 9SH UK; 2 Safe Sociable London Partnership, Peckham Levels, Level 4, 95A Rye Lane, London, SE15 4ST UK; 3 Southwark Council, 160 Tooley St, London, SE1 2QH UK; 4 London Healthy Place Network, London, UK

**Keywords:** England, Alcohol, Licensing, Public health, Ethnography, Process, Local government, Mixed methods

## Abstract

**Background:**

Public health in England has opportunities to reduce alcohol-related harms via shaping the availability and accessibility of alcohol through the licensing function in local government. While the constraints of licensing legislation have been recognised, what is currently little understood are the day-to-day realities of how public health practitioners enact the licensing role, and how they can influence the local alcohol environment.

**Methods:**

To address this, a mixed-methods study was conducted across 24 local authorities in Greater London between 2016 and 17. Data collection involved ethnographic observation of public health practitioners’ alcohol licensing work (in eight local authorities); a survey of public health practitioners (*n* = 18); interviews with licensing stakeholders (*n* = 10); and analysis of public health licensing data from five local authorities. Fieldnotes and interview transcripts were analysed thematically, and quantitative data were analysed using descriptive statistics.

**Results:**

Results indicated that some public health teams struggle to justify the resources required to engage with licensing processes when they perceive little capacity to influence licensing decisions. Other public health teams consider the licensing role as important for shaping the local alcohol environment, and also as a strategic approach for positioning public health within the council. Practitioners use different processes to assess the potential risks of licence applications but also the potential strengths of their objections, to determine when and how actions should be taken. Identifying the direct influence of public health on individual licences is challenging, but the study revealed how practitioners did achieve some level of impact, for example through negotiation with applicants.

**Conclusions:**

This study shows public health impact following alcohol licensing work is difficult to measure in terms of reducing alcohol-related harms, which poses challenges for justifying this work amid resource constraints. However, there is potential added value of the licensing role in strategic positioning of public health in local government to influence broader determinants of health.

**Electronic supplementary material:**

The online version of this article (10.1186/s12889-018-6306-8) contains supplementary material, which is available to authorized users.

## Background

Addressing health and social harms related to the consumption of alcohol has been of considerable public health concern for some decades [1]. While much attention has been paid to addressing alcohol consumption behaviours at the individual level, there is increasing recognition of the broader determinants of alcohol harms that arise from the environments in which alcohol is purchased and consumed [2]. There is growing evidence of the effectiveness of population-level interventions which shape the availability and accessibility of alcohol to reduce health and social harms including alcohol-related crime and anti-social behaviour as well as alcohol-related injuries and hospital admissions [3, 4]. This paper explores the possible contributions of public health practice to these mechanisms. We focus on the opportunities for public health practitioners (PHPs) in England to shape the provision of alcohol in local areas through licensing functions within local government, highlighting the situated processes, influences and outcomes of this role for PHPs.

The (re)positioning of public health into local government in England and Wales in 2013 was viewed as creating new opportunities for closer working between public health and other agencies, with focus on the broader social and environmental determinants of health [5, 6]. One example of this is the local alcohol environment which public health, alongside other agencies, can now seek to influence via the local government function of licensing premises to sell alcohol. The changes to the 2003 Licensing Act of England and Wales in 2011 [7] saw ‘health authorities’ designated as a ‘responsible authority’ within the licensing process, with a statutory right to review and make recommendations on alcohol licence applications [8]. Following the 2013 move into local authorities (LAs), public health teams undertake this licensing role alongside other ‘responsible authorities’, including agencies such as licensing, environmental health, trading standards, police and planning. There are hopes that through this new role public health teams will be able to help shape the local context of the availability and accessibility of alcohol [9], with potential to reduce alcohol-related health and social harms [3, 4]. What is not well understood is how this work is conducted: the processes and practices engaged as PHPs seek to contribute to the licensing function, and the extent to which public health can influence licensing decision making. In this paper we examine enactment of the public health licensing role across LAs in Greater London.

Public health teams can influence the alcohol licensing process in two ways. The first is through helping to develop local alcohol licensing policy, such as the mandatory Statement of Licensing Policy (SLP) [10]. This sets recommendations for licensing in the local area, and can designate cumulative impact policies (CIPs), which place additional restrictions on granting new licences in designated areas with ‘saturation’ of alcohol provision and related social harms [9, 11]. The second is contributing to assessment of licensing applications. Under the 2003 Licensing Act of England and Wales [12] applications to sell alcohol (for on- or off-premises consumption) will be granted unless objections (or ‘representations’) against the application are made, which are then considered and decided upon by the licensing sub-committee, comprising locally-elected council members. Under the Act, representations against applications for new licences or for variations to existing licences, or calls for reviews of existing licences, can be made only in reference to one or more of the four licensing objectives: i) prevention of crime and disorder, ii) protection of public safety, iii) prevention of public nuisance, and iv) protection of children from harm.

As none of the licensing objectives are explicitly health-focused, public health teams must frame their representations and recommendations for licence applications to licensing sub-committees in terms of other kinds of potential harm posed by a premises. Recent research has highlighted the challenges faced by some in attempting to align public health priorities with the licensing objectives’ focus on crime, safety and nuisance [13, 14]. Furthermore, in Scotland, where a fifth licensing objective to protect and promote public health was introduced in 2005, research indicates continuing challenges faced by PHPs in using population-level health data to support their representations against licence applications [15]. It appears that while the alcohol licensing role holds potential for public health to help reduce harms from the local alcohol environment, the reality of having effective influence over licensing decision-making remains difficult.

Recent literature on the positioning of public health in local government has highlighted the potential for conflicts around priorities, knowledge, and decision making – the ‘epistemologies of practice’ [6] – as PHPs attempt to work in new ways with a range of different non-health stakeholders and agencies [5, 16]. Furthermore, the organisational culture of LAs is considered to present new kinds of dynamics, hierarchies and political relationships previously unfamiliar to PHPs working within the health system pre-2013 [16]. These changes, coupled with the new role for PHPs to engage with the alcohol licensing process, give rise to important questions about how practitioners negotiate the expectations of conducting licensing work, within the context of LAs [17]. While guidance for this role exists, for example from Public Health England [18], there is very little known about the actual strategies and practices employed by PHPs, and how these shape and are shaped by the contextual dynamics of working in LAs. Moreover, little is understood about what kinds of influence public health can actually have over licensing decision making, and what might constitute ‘success’ within the public health licensing role.

Drawing from recent literature critically examining how evidence-based policy making is *done* in practice (see for example [6, 17, 19]), this paper describes a mixed methods study which aimed to explore the public health alcohol licensing role and how it unfolds within the day-to-day context of LAs in Greater London. With an emphasis on *“the social and material doing*” ( [20]: 122), of PHPs’ alcohol licensing work, we examined the processes, practices and impacts of public health contributions to licensing decision making. We highlight these to draw conclusions about how the public health licensing role can be better supported to strengthen influence over the local alcohol environment and reduce health and social harms.

## Methods

### Study design and context

We designed a multi-component, mixed methods study to explore the role of public health in alcohol licensing in Greater London, conducted between September 2016 and December 2017. The study methods comprised: i) ethnographic observation of PHPs’ day-to-day licensing work; ii) a survey of PHPs to explore licensing workload, approaches and perceptions of influence; iii) focus group discussions (FGDs) with PHPs, other responsible authority practitioners and other licensing stakeholders; iv) interviews with licensing stakeholders; and v) analysis of 9 months of routine public health licensing data from multiple local authorities. Reflecting the framing of the study in terms of understanding the reality(ies) of the public health licensing role, and how it was *“conceived and delivered in everyday practice*” ( [17]: 81), here we present findings drawn primarily from the ethnographic observation, supplemented by analysis from the survey, interviews and routine data. Further findings from the survey and FGDs on perceptions of how to strengthen public health contributions to licensing, drawing on data from the survey and FGDs have been reported elsewhere [14].

### Study sample

Sampling of local authorities within Greater London was conducted in different ways for the different forms of data collection, reflecting the aims of each method. These sampling strategies for the four sources of data represented in this paper are summarised in Table 1 below. Overall, 24 of the 33 LAs in London (including City of London Corporation) participated in one or more component of the study; see Table 2.

**Table 1:** Summary of data collection methods and sampling approaches for whole mixed-method study

Data collection method	Summary of method	Sampling approach	Participants / respondents	
			Total number	Details
Ethnographic observation	Observation of PH practitioners’ licensing work over period of 8 to 10 weeks in each LA.	*Purposive:* to capture range of inner and outer London boroughs; those public health teams known to be more active in licensing process	8 local authorities (multiple observations over 8 weeks per LA, on average).	5 inner London boroughs, 3 outer London boroughs.
Survey	Online questionnaire with questions on licensing workload, priorities, approaches, relationships with other RAs and perceptions of influence.	*All* 33 LAs in Greater London approached to find appropriate public health contact to complete survey.	18 responses (16 PH practitioners representing 18 LAs^a^)	11 inner London boroughs, 7 outer London boroughs.
Interviews	To explore perceptions of the public health role in licensing	*Purposive*: to capture range of licensing stakeholder perspectives, including from LAs with little public health licensing activity.	10 participants	3 senior public health practitioners; 1 trading standards practitioner; 2 licensing practitioners, 1 police licensing officer; 1 regulatory services manager; 1 councillor and 1 barrister.
Routine data analysis	To identify and compare numbers of alcohol licence applications received, actions taken and outcomes reported by public health teams.	*Purposive:* identified 5 LAs actively engaged in licensing work and regularly taking action on applications.	9 months’ data from 5 local authorities	5 inner London boroughs.

^a^Two PH practitioners worked across two LAs each, so responded twice (once for each LA)

**Table 2:** Summary of participating local authorities and the different components of the study in which they were represented

Participating local authority		Component of study				
	Inner/outer London	Ethnography	Survey^a^	FGDs^a^	Interview	Routine data
LA01	Inner	Y	Y	Y	Y	Y
LA02	Inner	Y	Y	Y	Y	Y
LA03	Outer	Y	Y			
LA04	Outer	Y	Y	Y		
LA05	Outer	Y	Y	Y		
LA06	Inner	Y	Y	Y	Y	Y
LA07	Inner	Y	Y	Y	Y	Y
LA08	Inner	Y	Y	Y	Y	Y
LA09	Inner		Y	Y		
LA10	Outer		Y	Y	Y	
LA11	Inner		Y	Y		
LA12	Outer		Y		Y	
LA13	Outer		Y			
LA14	Inner		Y			
LA15	Inner		Y	Y		
LA16	Outer		Y	Y		
LA17	Inner		Y		Y	
LA18	Inner		Y	Y		
LA19	Outer				Y	
LA20	Outer			Y		
LA21	Inner			Y		
LA22	Outer			Y		
LA23	Outer				Y	
LA24	Inner			Y		
Total:	I = 13	8	18	17	10	5
	O = 11					

^a^Some participants worked across more than one local authority, so participated for multiple LAs

### Ethnographic observation

To understand the day-to-day realities of public health alcohol licensing work, ethnographic observation of PHPs’ work, informed by traditions of organisational ethnography [21], was conducted in eight LAs over periods of between six and 12 weeks. The amount of time and frequency spent observing this practice varied according to the workloads of each practitioner, but typically involved one or two observations per week, lasting between two and 3 hours each; in total, ranging from 12 h to more than 30 h in each LA. The observations involved the PHP(s) (usually one, occasionally two) in each team responsible for screening and taking action on alcohol licence applications. Observations usually involved the researcher (JR) sitting with the PHP as they worked through licence applications, explored data sources and drafted representations against applications where appropriate. Although the researcher did not participate directly in these tasks, there was ongoing dialogue between researcher and practitioner during the observations talking about the work, concerns with applications, why certain actions were being taken, and discussing licensing work more generally.

In addition to this desk work, ethnographic observations were conducted (with permission) during meetings and other interactions relating to public health alcohol licensing work, such as regular ‘responsible authority’ meetings held in some LAs. These meetings enabled understanding of how public health engaged with and was positioned relative to other council practitioners undertaking licensing work. Finally, the researcher undertook observations of licensing sub-committee hearings that occurred during the data collection period and which involved contributions from public health, for example if they had submitted a representation against an application. Fieldnotes were recorded during and after all observations and interactions during the ethnographic data collection, and provided opportunities for the researcher to reflect on the unfolding insights and their positionality.

### Survey

The survey aimed to capture information about PHPs’ approaches to alcohol licensing work and the amount of work undertaken. While attempts were made to identify the relevant public health contact involved in alcohol work in all 33 Greater London LAs, it was only possible in 28 LAs. An invitation to participate and a link to an online questionnaire (see Additional file 1) were sent directly to the relevant PHPs identified across the 28 LAs, and follow up prompts were sent as necessary. Practitioners from 18 LAs completed the survey (18 responses in total), a response rate of 64% of those invited (54% of all London LAs). Proportionally more inner London LAs completed the survey than outer London LAs; 11 out of 13 inner London LAs participated, compared with 7 out of 20 outer London LAs. The online questionnaire took around 15 min to complete and included questions on the amount of alcohol licensing work undertaken by the public health team, the frequency of different kinds of actions taken on applications, and the resources used to justify representations. Participants were also asked to rate their perceived influence on the licensing process.

### Interviews

Ten semi-structured interviews were conducted to explore a range of stakeholders’ perspectives on the role of public health in alcohol licensing and the challenges and opportunities posed by this role. Efforts were taken to identify multiple types of licensing stakeholder from across different London LAs, including those where little or no public health alcohol licensing work was happening at the time. Participants included public health alcohol leads; licensing, trading standards and environmental health practitioners; enforcement services managers; police licensing officer; a council solicitor; and a local councillor sitting on the licensing committee. Interviews were conducted usually at participants’ workplaces, at LSHTM or a local café, or by telephone in the case of one participant. Questions were modified according to participants’ individual role and professional context; see Additional file 2 for a sample interview topic guide. They were all audio-recorded with participants’ permission, lasting between 45 and 90 min, and were transcribed verbatim.

### Routine licensing data

The data routinely recorded by public health teams on alcohol licence applications received and actions were collected, with permission, from five LA authority areas over a 9 month period (January to September 2017). These LAs were sampled purposively, to include areas known to have active public health involvement in licensing, including regularly taking action on applications. This comprised information on a total of 571 applications (including new licence applications, variations and reviews). The key information extracted for the analysis included: type of application; type of premises; if in a cumulative impact zone; whether action taken and if so, what type (negotiation, representation); and the outcome of the action taken. For almost all data sets there were missing data, particularly in terms of the outcomes of actions taken, and this was supplemented where possible with information from the LAs’ licensing registers about the outcome of applications.

### Analysis

The ethnographic fieldnotes and interviews were analysed using a thematic analysis approach. Separate coding frameworks were developed by JR and MM for each source of data (fieldnotes and interview transcripts), but with regular comparison and dialogue between the two as analysis progressed. Fieldnotes and transcripts were coded line-by-line in Nvivo 11, with codes gradually being grouped into categories reflecting broader themes. Key themes that were explored in both data sets included: the position and role of public health, and uses of data and evidence.

The quantitative data from the survey and routine licensing data were subjected to descriptive statistical analyses in Excel, reflecting the small sample size of each which precluded any meaningful tests of statistical significance. Final interpretation of the themes and survey data was supported by the other authors.

## Results

From across all four sources of data, three key areas of interest were identified, relating to the processes, practices and influence of public health licensing work. These include: i) why licensing work is or is not done by public health; ii) the approaches and strategies employed in licensing work; and iii) defining and measuring impact of public health in licensing work. The predominant focus of the results is the day-to-day work of dealing with premises licence applications and reviews. While many participants talked about the role of public health in contributing to broader licensing policy, this work happens much less regularly and did not arise in any of the ethnographic observations during the fieldwork period.

### Why licensing work is or is not done by public health

**Fig. 1:**
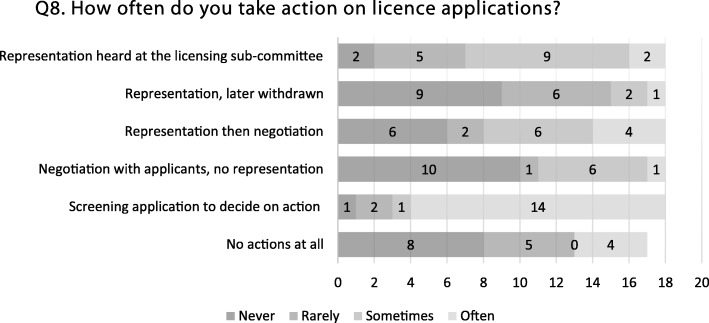
Bar chart illustrating the frequency of different types of actions taken on licence applications reported by survey respondents (n = 18)

The survey results indicated that almost all PHP respondents (17/18) undertake at least some work in relation to alcohol licence applications. However, the type of action taken varied considerably across the sample, from only screening applications, up to regularly making representations that are heard at licensing sub-committees (see Fig. 1). This was supported by the average number of person-hours respondents reported spending each week on alcohol licensing work, ranging from 0 to 2 h (10/18 respondents) to 6 or more hours (2/18 respondents). The survey’s sample likely reflected those public health teams more actively involved in alcohol licensing work, given that it was not possible to locate an alcohol lead in five of the 33 London LAs. Therefore, it is possible that there were more teams across London where little or no regular alcohol licensing work was taking place at the time of the study.

The ethnographic observation and interviews provided opportunities to understand the value that PHPs placed on alcohol licensing work and, from the interviews, explanations for why some PHPs did not contribute much or anything to the licensing process. While the main focus of acting on individual licensing applications was framed in by PHPs as attempting to shape the availability of alcohol, many also spoke of broader, more strategic reasons for contributing to the licensing process. One PHP described her licensing work as part of a balance between the “*micro focus*” and the “*macro focus*” of addressing alcohol-related harms, through both drinking behaviours and wider determinants. Other PHPs described licensing work more as a mechanism for positioning public health within the LA, to help ‘embed’ public health values and priorities across council work:

*“[PH practitioner] said . . . the DPH sees [licensing work] as important for the broader strategic direction of public health within local authorities” *(Ethnographic field note, LA-02).

Related to this, a few PHPs involved in licensing work indicated that while they did not feel they had much influence over decision making on individual licence applications, they thought it was important strategically to be “*part of the process*” and to be seen to be “*making connections*” with other directorates in the council. This was reinforced by findings from the survey; more PHPs (16/18) stated that ‘Increasing understanding of public health perspectives and values’ across the council was a priority for alcohol licensing work than ‘making a representation that is upheld at licensing sub-committee’ (14/18 respondents).

Conversely, some PHPs reported doing little or no regular alcohol licensing work including two respondents to the survey who stated that they never screen alcohol licence applications. There appeared several explanations for this; for some, it was a lack of capacity to undertake screening and actions on applications alongside their other public health duties such as commissioning alcohol treatment services. This indicated that alcohol licensing was not considered high enough a priority in comparison with other areas of public health work. For others, they did not think public health can have much (or any) impact through making licensing representations and so had either never tried to undertake this work, or had stopped after seeing no influence:

*“So um, to be frank, I think four or five applications where I went and presented evidence, um, and it just wasn’t successful, um, and so after that, er, to be frank, we haven’t tried. So I think the last one we did was probably about two years ago. . . I don’t even look at them, to be honest.*” (Interview with public health alcohol lead)

Hence, the perceived capacity to have influence on licensing decision making appeared to be a strong factor shaping some PHPs’ decisions around whether to process and take action on individual licence applications. Further discussion of the perceived status of public health within the licensing process is presented elsewhere [14].

### Approaches and strategies employed in licensing work

Observed through the ethnographic work were a range of approaches undertaken by PHPs as they engaged with alcohol licence applications and made decisions regarding whether, and how, to take action on them. These strategies reflected a number of different processes, including conceptualisation of health-related priorities relating to how and where alcohol is sold; engagement with and interpretation of different sources of data; and perception of likelihood of successful outcomes of any public health input.

#### Identifying potential health-related issues

PHPs’ licensing work typically involved ‘screening’ new licence applications (or applications for reviews of existing licences), to identify any concerns with the application that required further investigation. In some LAs, the PHPs had a set of explicit criteria or priorities against which they screened applications, often presented as column headings on a spreadsheet used to record information on each licence application received. In other LAs, the criteria seemed more implicit and less fixed, identified by the researcher asking practitioners to explain how they were screening the applications. There were some similarities across areas in terms of the types of issues or features of a licence application that would be flagged by PHPs as potentially problematic. These usually reflected issues around the accessibility and availability of alcohol, for example through the hours of sale or type of premises (such as ‘vertical drinking’ venues such as bars), or concerns about existing levels of harm in particular areas of high density of alcohol outlets (such as in cumulative impact zones, CIZs).

*“[PH practitioner] started completing the log, noting the dates of the application in and deadline, the name and premises name, the address, that they want both on and off sales, and that it’s a new licence application. When she came to the hours section, she said “that’s what I have a problem with, the 08.00”.*” (Ethnographic field note, LA-04).

Though not always explicitly articulated, these issues appeared to reflect assumptions about the likelihood of increased health and social harms resulting from premises contributing to the availability of alcohol.

#### Engagements with data to assess risk of harm

Following this initial screening, many PHPs would consult a range of sources of information or data to explore in more detail issues relating to those proposed (or existing) licensed premises flagged as potentially problematic. The purpose of this, as observed through the ethnographic fieldwork, appeared to be to assess further the level of risk posed by the licence application but also to assess the robustness of a public health objection (or representation) against the application. This assessment helped PHPs decide whether it would be valuable – from a public health perspective - to submit a representation.

The data sources regularly consulted by PHPs included databases and data tools comprising police, transport and ambulance data that recorded alcohol-related crimes, ambulance call-outs and injuries, and other relevant incidents such as serious assaults. These sources were externally produced, for example, data tools developed by Safe Sociable London Partnership (safesociable.com), and the ‘Safe Stats’ databases provided by the Greater London Authority (london.gov.uk) or developed in-house from external sources of data. These were typically searchable at the postcode level, and / or produced comparisons between neighbourhoods or wards for selected variables. The ethnographic observation helped illustrate how PHPs practitioners engaged with these sources to identify levels of risk of alcohol-related harm connected to the location in which a premises was situated:

*“[PH practitioner] said he’d take the postcode and put it into the traffic light worksheet . . . He looked up where the premises is and then at the ward data . . . Under the ambulance data, he noted that the ward in which [premises] is located was 3*^*rd*^ *out of 19 for sexual assault, and . . . and identified that it was low for underage illness, and said that it looked like the premises was in a “not very alarming area”.*” (Ethnographic field note, LA-03).

This ‘spatialisation’ of risk occurred through other sources of data too, including different types of maps, either produced in-house or available externally for example from the police. Some practitioners consulted maps to identify deprivation in the local area, reflecting an assumption that alcohol-related harms are suffered more by those who are more deprived. Maps were also used on occasion to identify the location of relevant services, such as schools or alcohol treatment centres, which might be negatively affected by the provision of alcohol through a new or existing premises, and also to identify the location of a premises relative to the boundaries of any cumulative impact policy zones.

As such, these varied sources of data and information provided mechanisms through which PHPs could assess the potential risk of a proposed or existing premises based on spatialised interpretations of the severity of recorded alcohol-related incidents in a locality, and burden on particular groups or services. Moreover, these engagements with data also appeared to inform whether public health action should be taken, based on an assessment of the perceived ‘strength’ of the data as evidence to support public health objections. More detailed examination of the values placed on different kinds of data and evidence by public health and other licensing practitioners are reported elsewhere (McGrath M, Reynolds J, Lock K: “If you start with liver disease, you're gonna get nowhere fast”. Framing public health knowledge and evidence in local alcohol decision-making, forthcoming).

#### *‘*Picking battles’: Strategies for taking action

There are several actions PH practitioners can take in their role as a responsible authority if they consider a licence application to pose risks including recommending refusal or revocation of a licence, recommending restrictions on the licence (for example reducing hours of sale or restricting to on-sales only), and / or recommending additional conditions for the licence (for example setting a maximum alcohol strength for beers and ciders to be sold). Through the study, how PHPs seek to take action and influence the decision on a licence application varied. Approaches taken reflected assessments by PH practitioners of likelihood of influencing a licence application and / or the licensing sub-committee’s judgement, as well as assessments of the time and resources available to inputting into acting on a licence application.

For applications considered problematic, some PHPs observed sought to negotiate with the applicant(s) first on details of the licence application (such as reducing hours of sale or adding conditions to the licence), before submitting a representation to the licensing sub-committee if negotiation was not successful. Others, however, would submit a representation, and either use that as a starting point to begin negotiations or not seek to negotiate at all. From the survey, 10 out of 18 PHPs stated they often or sometimes submit a representation and then negotiate, compared with seven out of 18 respondents often or sometimes negotiating without submitting a representation. Deciding whether (and when) to negotiate with applicants appeared to reflect perceived severity of the issues connected with an application, as well as capacity to submit a full representation. In one LA, the PHP described during ethnographic observation an approach that distinguished between applications with *“minor”* and “*major”* concerns. A template letter requesting additional conditions would be used to negotiate with applicants of *“minor”* applications, whereas a full representation would be prepared and submitted to the licensing sub-committee for *“major”* applications. This approach was described as helping to overcome a lack of capacity within the public health team; the PHP stated it was not always “*worth the investment”* to commit time to submitting full representations for lower risk applications.

The analysis of routine public health data from five LAs indicated that successful negotiation between public health and applicants occurred for 43.9% of the applications on which public health took some form of action, meaning that full representations were either not submitted or were withdrawn. Seeking to negotiate terms of a licence with applicants prior to (or instead of) submitting a full representation appeared to be a mechanism for PHPs to achieve some level of influence (if not the full desired outcome) while avoiding what was considered by some to be the ‘risky’ space of the licensing sub-committee hearing. One PHP felt that the licensing sub-committee in her LA did not often make decisions in line with public health representations, and that she was *“more successful”* when negotiating directly with applicants. A few PHPs perceived additional ‘risks’ in submitting full representations to the sub-committee, including negatively affecting relationships with the sub-committee and other RAs if public health representations were considered to be unnecessary or not clearly justified. One PHP indicated during the ethnographic observation that she had been advised by her line manager on several occasions not to put in representations against applications, as a way of “*managing the relationship with licensing going forwards”*.

This perspective was reinforced by other licensing stakeholders, including in an interview with a councillor and member of a licensing sub-committee who indicated that she preferred to see RAs (including public health) trying to reduce problems posed by licence applications, rather than submitting representations requesting refusal:

*“[recommend what the applicant] can do to mitigate what the problem is rather than just refusing the application. So for example if your issue is around street drinkers having super strength lager you can put conditions on licence that bans them selling super strength lager which is probably more desirable than just banning the business from existing.”* (Interview with councillor).

Hence, the approaches taken by PHPs reflected strategies of ‘picking battles’; seeking to balance attempts to influence alcohol licences to reduce harms with the resource implications and potential risks connected to submitting full representations to be heard at licensing sub-committee.

### Defining and measuring public health ‘impact’

Through the study there emerged different ways in which the influence of public health in the licensing process was conceptualised, and what ‘success’ might look like from a public health perspective.

#### Identifying the public health contribution

The lack of health licensing objective in England has been identified as potentially limiting the influence of public health on licensing decisions [13, 14]. In this study, many PHPs described occasions where their representations against licence applications had not been ‘successful’ in influencing the licensing sub-committee’s decision, and some other licensing stakeholders perceived public health to have little scope for influence:

*“I don’t think [public health] have any powers. . . I mean anyone can comment [on a licence application]. They have no, no more power than a resident in terms of saying these are the impacts that this activity could potentially cause.”* (Interview with licensing officer).

However, through the ethnographic observation and analysis of routine data, there was some indication of the influence of public health on individual licence applications, particularly (as discussed above) through negotiating additional conditions and restrictions with applicants outside the sub-committee sphere. From analysis of routine data from five public health teams, there was some level of public health ‘impact’ recorded on an average 84.1% of the applications on which PH practitioners took action. In addition to the successful negotiation in 43.9% of applications on which public health action was taken, 21.3% resulted in refusal or revocation of the licence, 14.9% in the licence granted with extra conditions or restrictions, and 4.0% resulting in the refusal of a variation to a licence. This measure of ‘public health impact’ is arguably a crude one, as it was not possible to identify from the data the extent to which all of the recommendations made by public health were upheld in the final outcome of an application. See Table 3 for a summary of the routine data analysis.

**Table 3:** Summary analysis of routine public health data on alcohol licence applications received by public health in 5 local authorities between Jan and Sept ‘17

	LA-01		LA-02		LA-06		LA-07		LA-08		Total	Mean
	n	%	n	%	n	%	n	%	n	%		
Total applications	145		188		42		128		68		571	95.2
Applications on which action taken^a^	48	33.1%	57	30.3%	10	23.8%	0	0%	4	5.9%	119	18.6%
Of which, formal representation made	11	7.6%	57	30.3%	10	23.8%	0	0%	4	5.9%	82	13.5%
No action taken	87	60.0%	129	68.6%	32	76.2%	127	99.2%	64	94.1%	439	79.6%
Unclear if action taken	10	6.9%	2	1.1%	0	0.0%	1	0.8%	0	0.0%	13	1.7%
Outcomes of PH actions taken												
Licence revoked / refused	0	0.0%	3	5.3%	3	30.0%	0	0%	2	50.0%	8	21.3%
Variation refused	2	4.2%	1	1.8%	1	10.0%	0	0%	0	0.0%	4	4.0%
Licence granted with restrictions	1	2.1%	10	17.5%	4	40.0%	0	0%	0	0.0%	15	14.9%
Successful negotiation^b^	40	83.3%	27	47.4%	2	20.0%	0	0%	1	25.0%	70	43.9%
No success (licence granted / upheld)	0	0.0%	3	5.3%	0	0.0%	0	0%	0	0.0%	3	1.3%
Application withdrawn	4	8.3%	6	10.5%	0	0.0%	0	0%	0	0.0%	10	4.7%
Unclear / unknown	1	2.1%	7	12.3%	0	0.0%	0	0%	1	25.0%	9	9.8%

^a^Action taken includes negotiation with applicant and / or submission of representation

^b^This includes successful negotiation on at least one, but not necessarily all public health recommendations

Furthermore, the extent to which the outcomes of licence applications can be attributed to public health *alone* is often difficult, given that actions may be taken by other responsible authorities (and sometimes residents) against the same application, and the weight attributed to each in the decision making by a committee is often not clearly documented:

*“[the licensing sub-committee’s verdict] highlighted the lack of credibility around the applicant’s statements about his relationship with the business and in relation to the incidents, and that they were concerned about the ability to manage the premises appropriately. As such, there was nothing specific about public health’s contributions in the written verdict, but [PH practitioner] was extremely pleased at the result, and felt that their efforts had paid off.”* (Ethnographic field note, LA-03).

However, this lack of attributable impact is not necessarily problematic. As reported elsewhere [14], collaborative working between public health and other responsible authorities was seen as valuable and influential for licensing work, and the ‘right’ outcome (however achieved) may be sufficient to justify public health contributions to the process.

#### Likelihood of ‘success’ with action on applications

From the ethnographic observation, a range of examples of public health influencing individual licences were identified. In a couple of LA areas, the PHPs stated that they thought their licensing sub-committees understood well the public health perspective and that when they made representations against licence applications, they would be considered carefully and appeared often to influence the committees’ decisions. One PHP said that although she sometimes felt constrained by a lack of health licensing objective, she had never “*come away with nothing”* from licensing sub-committee hearings when she had submitted representations. In other LA areas, however, PHPs appeared more doubtful of their ability to influence decisions, meaning more effort was made to negotiate with applicants (as discussed above) but also that ‘successes’ at licensing sub-committee hearings were more noticeable and celebrated. In one LA, the public health team shared news of their *“win*” at a licensing sub-committee hearing for the review of a licence following what was considered influential input by one PHP:

“*I received an email from [PH practitioner] saying he’d been told the committee had deliberated for only 10 minutes and had decided to revoke the license. He said he’d been told that the point [made by public health] about the potential harm to children through noise and disturbance had been quite influential in the decision making. . . [PH practitioner] seemed very pleased by the outcome, describing it as a “win” and saying it would be useful to write this up and see if the child protection point can be used again in the future.*” (Ethnographic field note, LA-02).

Here, the use of the PHP’s expert opinion on evidence around sleep and children’s well-being was explicitly identified as influential in shaping the committee’s decision to revoke the licence, and was considered valuable for use in future public health representations and arguments.

Furthermore, there were indications that public health actions might be more successful for certain types of licence application and premises. Although some licensing stakeholders doubted the capacity for public health to influence licensing outcomes, others were able to recall ‘successful’ inputs by public health for specific types of premises:

*“I think they’re probably more effective in off licences. From . . . looking, I think they’re far more effective on off licences because they, and already they’re looking at, you know, percentage of alcohol by volume, single sales, single can sales, unit pricing and obviously the off sales aspects.”* (Interview with police licensing officer).

Here, the police licensing officer appears to imply that features of the way alcohol is sold in small shops with off-premises licences as more compatible with public health concerns about types of ‘problem’ drinking behaviours.

#### Broader influences on licensing

Although the main focus of this study was on the day-to-day licensing work undertaken by public health, there were some indications of public health influence on licensing more widely. This included shaping other responsible authorities’ approaches to assessing risks of licensing applications. For example, in one LA, a PHP stated that she had seen the licensing team “*pay more attention to hours*” requested in licensing applications following several public health representations focusing on the risks from early sales of alcohol.

There were also perceptions among PHPs and other licensing stakeholders that the population perspective characteristic of public health was well suited to influencing area-wide policies such as each local authority’s Statement of Licensing Policy and cumulative impact policies. One PHP suggested that they had made more impact on the availability of alcohol through shaping the content of wider policies than by acting on individual licence applications:

*“we have influenced the policy and influenced where the cumulative impact zones are. . . I think that’s where we added, you know, we made the most difference.”* (Interview with PH practitioner).

In one LA participating in the ethnographic observation, the influence of the public health team on specific recommendations around hours of alcohol sales in the SLP was noted by practitioners, and in turn, the SLP was used regularly by the PHP to justify representations against applications requesting late hours of sales.

However, PHPs also acknowledged the political nature of the setting of area-wide policies such as the SLP which limited their potential influence. There was understanding that public health values for reducing harms from alcohol availability may be perceived to be at odds with other council strategies, for example to support the local economy and regenerate deprived areas. In one LA, a PHP expressed concern about the weight placed by the licensing committee on local businesses’ views about licensing policy over *“the evidence”* of increasing alcohol-related harms in some areas. She said she thought that when the SLP is due to be revised, the committee would *“bottle it again”* in relation to public health recommendations to extend cumulative impact areas. Hence, while influencing broader licensing policies was generally considered to be a productive way for public health to have impact on the availability and accessibility of alcohol, there were clear limitations to this impact posed by the wider contextual and political challenges of implementing and managing policy.

## Discussion

Public health has an important role in reducing health and social harms from alcohol by acting on the broader alcohol environment and the policies and practices which shape it. Opportunities for public health practitioners to enact this role have increased in England since changes to licensing legislation and the (re)positioning of public health in local government [13]. However, little attention has so far been paid to the day-to-day realities of performing the public health licensing role in this setting. Understanding the processes and adopted by PHPs in navigating new expectations and responsibilities for influencing licensing decision making is important for determining how best to strengthen actions on the wider determinants of alcohol-related harms. This paper presented an in-depth study of the public health role in alcohol licensing processes in local authorities in Greater London, to identify the ways in which PHPs seek (or do not seek) to influence decisions on alcohol licence applications.

Through mixed methods we were able to draw out detailed accounts of the *“actualities*” [17] of screening, assessing and acting upon licence applications, and the various strategies and tools practitioners employed to do so. We also generated a broader understanding of how these practices were shaped – and often constrained – by the relational context in which they occurred, influenced by relationships with other licensing stakeholders, the availability of resources and capacity, and strategies to prioritise and maximise the ‘gain’ from public health work in an era of budget constraints. The process of assessing the potential risks posed by licence applications, through engagement with different data sources, also represented a process of assessing the likelihood of ‘success’ of a public health objection, as a means of ‘picking battles’. PHPs’ perceptions of (some) data sources as limited in their ability to act as persuasive ‘evidence’ of the (potential) harms of a premises also contributed to the assessment of whether to take action on an application. This corresponds with reflections from other contexts, such as Australia, on the challenges of converting epidemiological data on harms relating to the availability of alcohol to practical recommendations for policy [22]. Furthermore, while the potential value of public health contributions to broader licensing policies (such as the Statement of Licensing Policy) was acknowledged by many in this study, echoing other recent research [15, 23], the political dimensions and timescales of the policy-making process appeared likely to restrict the capacity for PHPs to have influence in this way.

However, the analysis of public health actions through use of routine data enabled understanding of the high levels of impact on licensing outcomes that some PHPs had when actions were taken. This was supplemented by more detailed case examples of successful actions taken by practitioners during the ethnographic observation. In particular, actions taken to negotiate with licence applicants were commonplace – and often successful – linked to perceptions of the ‘risks’ (of time and effort required, compared with likely outcome) of having representations against applications heard at licensing sub-committees.

These findings highlight the multiple strategies employed within public health practice in a multi-disciplinary context where health is not always the foremost priority [24]. This is perhaps more acute in the context of alcohol licensing where PHPs have responsibility to review and act on licence applications where appropriate, but lack legislative authority in the form of a health-oriented licensing objective [13]. This strategising also reflects the uncertain and challenging context of conducting public health within reduced budgets and capacity, and concerns around how and where best to ‘invest’ public health time and resources to bring about effective benefits for the health of the local population [25]. Perhaps one of the key challenges of the alcohol licensing function is the difficulty in identifying ‘real’ impact on alcohol related harms following public health action on individual licence applications, to justify the effort and time required to engage with the process. In a public health landscape foregrounding outcome measures and demonstrable cost-effectiveness [26], it is understandable that some PHPs do not consider involvement in the alcohol licensing process a worthwhile trade of efforts required for (perceived) impacts gained.

However, what this study also highlights is the range of other ways in which ‘success’ of the public health licensing function was conceptualised by practitioners. Instead of (or as well as) influencing individual licence applications, and the harms linked to the availability and accessibility of alcohol, many PHPs saw licensing work as valuable for positioning public health in the local authority. Licensing work was conceptualised as facilitating engagement with other agencies, and for promoting the perspectives and priorities of public health more generally across the council. This suggests that the alcohol licensing function, when conducted regularly and through engagement with other responsible authorities, might be considered a means for a different end, of establishing public health values across multiple different areas of local government work. This perspective echoes the ‘health in all policies’ agenda [27], and the importance of bringing health-related values to the forefront of decision making across multiple areas of policy making that reflect the broadest determinants of health [28].

Productive public health engagement with the alcohol licensing process under the current legislative framework in England appears to necessitate relationship building with other responsible authorities [14], and skills in accessing and communicating different types of data in line with the expectations of the local licensing sub-committee and licence applicants. This echoes analyses of local alcohol licensing and policy processes in similar international contexts such as Australia and New Zealand, which have highlighted the importance of collaborative and partnership working to effect change within political contexts with competing interests [29, 30]. Given that positive impacts of public health actions on the local alcohol environment are challenging to identify and attribute, it is easy to see this work may not be considered justifiable by senior public health practitioners in the face of more easily measurable health priorities. However, it appears the licensing role has an important place in the broader work of positioning public health in local government, and how relationships with other stakeholders can be mobilised to influence decision making affecting the wider determinants of health [31].

### Limitations

The sampling for this study reflects a bias towards public health teams who were more actively engaged with alcohol licensing, although we did seek views from LAs with little or no public health licensing input. The sample sizes for the routine data analysis and survey were too small to enable tests of statistical significance, but reflect the pragmatics of conducting applied research in these contexts. Furthermore, with a focus on local authorities across Greater London, the results of this study are not easily generalisable to the broader England context or other places with similar legislative structures. However, the considerable variety across the LAs included in the study in terms of demographics, politics, night-time economies and levels of deprivation facilitates the transferability of the results. These can be useful for informing discussions in other areas around how best to direct limited public health resources to tackling alcohol-related health and social harms, and promote public health values more broadly in local government.

With the focus predominantly on the work of screening and acting on individual licence applications this study can only draw limited conclusions about the role and influence of public health in broader alcohol policy making at the local level. We recognise there is still more scope for understanding the day-to-day realities of undertaking this policy-making work from the public health perspective, in terms of the strategising required to help reduce harms to health from the local alcohol environment.

## Conclusions

This study highlighted the range of processes and practices undertaken by PHPs as they sought to negotiate and enact the role of ‘responsible authority’ within the alcohol licensing process in local government. With current constraints on public health capacity and resources at the local level, it is challenging for some PHPs to justify alcohol licensing work when it is difficult to demonstrate making impacts on the availability and accessibility of alcohol, and on related harms. However, this study suggests an added value for licensing work through the strategic alignment of evidence and relationship-building, to facilitate the positioning of public health within local government functions to influence wider determinants of health.

## Additional files

Additional file 1: Alcohol Licensing in Local Government. Copy of online questionnaire. (PDF 125 kb)

Additional file 2: Sample semi-structured interview topic guide for public health alcohol lead. Sample topic guide used for semi-structured interviews. (PDF 342 kb)

## Abbreviations

CIP: Cumulative impact policy
LA: Local authority
PHP: Public health practitioner
RA: Responsible authority
SLP: Statement of licensing policy
