# Supplementary material for: Processes, practices and influence: a mixed methods study of public health contributions to alcohol licensing in local government
Source: BMC Public Health. 2018 Dec 18;18:1385. doi: 10.1186/s12889-018-6306-8 (PMC6299525; doi:10.1186/s12889-018-6306-8)
Supplement: Supplementary file 1 — Alcohol Licensing in Local Government. Copy of online questionnaire. (PDF 125 kb) [file 12889_2018_6306_MOESM1_ESM.pdf]

Alcohol licensing in local government

Page 1: Study information and consent

Exploring influences on the public health contribution to alcohol licensing processes in local government

We are a group of researchers from London School of Hygiene & Tropical Medicine conducting a research study to explore the range of factors that shape how public health practitioners seek to influence alcohol licensing decisions, and the outcomes of this. This study is part of a broader programme of research funded by the NHS National Institute for Health Research looking at how to support local government work to tackle alcohol-related harms.

We would like to invite you to participate in this study by completing the following questionnaire on your current work and practice in relation to alcohol licensing. The questionnaire is designed to capture important information about how public health practitioners in the London area approach alcohol licensing work, and when and how they make decisions on licence applications. It should take around 10 to 15 minutes to complete this questionnaire.

The information captured in this questionnaire will be analysed alongside data from other components of the study to generate an in-depth picture of the range of influences on public health practice in relation to alcohol licensing in local authorities in London. All data will be kept confidential. Your name will not be used or shared, and your local authority and any other identifying details will be anonymised in all reporting of the study findings.

If you have any questions about this questionnaire or the wider study, please contact Jessica Engen at London School of Hygiene & Tropical Medicine - jessica.engen@lshtm.ac.uk, 020 7927 2380 or Joanna Reynolds - Joanna.reynolds@lshtm.ac.uk, 020 7927 2023.

1. By completing this questionnaire, I understand that I give my consent for my responses to be included in the study described above ✱ *Required*

Page 2: Your details

2. Your name (optional)

3. What is your job title?

4. For which Local Authority(ies) do you do alcohol licensing work? (This will be anonymised) ✱ *Required*

4.a. If you work across more than one Local Authority, for which Local Authority will you answer this questionnaire? *Please choose only one* (This will be anonymised)

5. How long have you been doing alcohol licensing work in a public health capacity?

- 0-6 months
- 7-12 months
- more than 12 months

6. On average, how many alcohol licence applications do you recieve each month in your Local Authority? (including new applications, variations and reviews)

- [ ] 0 - 5
- [ ] 6 - 10
- [ ] 11 - 15
- [ ] 16 - 20
- [ ] 21 +

7. On average, how much time is currently spent on alcohol licensing work for public health within the council (person hrs / week)?

- [ ] 0 - 2
- [ ] 3 - 5
- [ ] 6 - 8
- [ ] 9+

Page 3: Action on alcohol licensing applications

8. What forms of action do you typically take on alcohol licence applications (including new applications, variations and reviews) and how often?

	Often	Sometimes	Rarely	Never
No actions at all	☐	☐	☐	☐
Screening application to decide on action to take	☐	☐	☐	☐
Informal action (negotiating conditions with applicant without submitting a representation)	☐	☐	☐	☐
Representation then negotiation (submitting a representation, then negotiating with the applicant before withdrawing the representation)	☐	☐	☐	☐
Representation, later withdrawn (submitting a representation, then withdrawing it for another reason, not due to successful negotiation)	☐	☐	☐	☐
Representation heard at the licensing sub-committee	☐	☐	☐	☐

9. By which criteria or priorities do you screen alcohol licence applications and decide whether to take action (either formally or informally)? *tick all that apply*

- ☐ Premises within cumulative impact zone / special policy area
- ☐ Requesting late hours
- ☐ Requesting early hours
- ☐ Vertical drinking / high volume drinking
- ☐ Off-licence
- ☐ Other off-sales

- [ ] Review of licence
- [ ] Other
- [ ] Do not screen alcohol licence applications

9.a. If you answered other, please specify

10. What type of information do you routinely record on applications your receive? *tick all that apply*

- [ ] Type of venue
- [ ] Type of licence requested
- [ ] Hours of alcohol sales
- [ ] Opening hours of premises
- [ ] Outcome of screening process
- [ ] Actions taken by public health
- [ ] Engagement with other Responsible Authorities
- [ ] Application taken to licensing sub-committee
- [ ] Outcome of public health action
- [ ] Outcome of licence application
- [ ] Other
- [ ] Do not record information on applications

10.a. If you selected Other, please specify:

Page 4: Making a representation

11. What sources do you draw on when **deciding whether** to make a representation on an application and how regularly do you use them?

	Always	Often	Occasionally	Never	N/A
Statement of Licensing Policy	☐	☐	☐	☐	☐
Safe Stats database	☐	☐	☐	☐	☐
Bullseye tool	☐	☐	☐	☐	☐
Traffic light tool	☐	☐	☐	☐	☐
Metropolitan Police crime maps	☐	☐	☐	☐	☐
Advice/guidance from other Responsible Authorities	☐	☐	☐	☐	☐
Systematic reviews	☐	☐	☐	☐	☐
In-house policy reports/strategies	☐	☐	☐	☐	☐
Other in-house data sources	☐	☐	☐	☐	☐
Published research papers	☐	☐	☐	☐	☐
Other policy reports (eg: from national government, PHE, NGOs etc)	☐	☐	☐	☐	☐
Other	☐	☐	☐	☐	☐

11.a. If you use in-house data sources, please specify

11.b. If you selected other, please specify

11.c. Any additional comments on deciding whether you make a representation:

12. What evidence and sources do you use when **writing** representations (when made) and how regularly do you use them?

	Always	Often	Occasionally	Never	N/A
Statement of Licensing Policy	☐	☐	☐	☐	☐
Safe Stats database	☐	☐	☐	☐	☐
Bullseye tool	☐	☐	☐	☐	☐
Traffic light tool	☐	☐	☐	☐	☐
Metropolitan Police crime maps	☐	☐	☐	☐	☐

Advice/guidance from other Responsible Authorities	☐	☐	☐	☐	☐
Systematic reviews	☐	☐	☐	☐	☐
In-house policy reports/strategies	☐	☐	☐	☐	☐
Other in-house data sources	☐	☐	☐	☐	☐
Published research papers	☐	☐	☐	☐	☐
Other policy reports (eg: from national government, PHE, NGOs etc)	☐	☐	☐	☐	☐
Other	☐	☐	☐	☐	☐

12.a. If you use in-house data sources, please specify

12.b. If you selected other, please specify

12.c. Any additional comments on writing representations:

13. Do you use any other sources, in addition to those marked above, when **preparing** for a licensing sub-committee hearing? Please specify.

14. What influences your decision **not** to take action on an application? Tick all that apply

- ☐ Screening process indicates application is low priority
- ☐ Lack of data to support representation
- ☐ Representation is not supported by the Statement of Licensing Policy
- ☐ Representation unlikely to be supported at licensing sub-committee
- ☐ No other Responsible Authority making representations
- ☐ Lack of time or capacity
- ☐ Other

14.a. If you selected Other, please specify:

15. How often do you feel you **do not** have time or capacity to take action on applications within the specified deadline?

Page 5: Priorities and capacity

16. What priorities do you consider to be important in Public Health licensing work?

	Very important	Quite important	Not very important	Not important at all
Agreeing conditions with applicant through negotiation before licensing sub-committee	☐	☐	☐	☐
Making a representation that is upheld at the licensing sub-committee	☐	☐	☐	☐
Working in partnership with other Responsible Authorities	☐	☐	☐	☐
Influencing alcohol licensing policy within the council to reflect public health values	☐	☐	☐	☐
Increasing understanding of public health perspectives and values more generally within the council	☐	☐	☐	☐
Contributing to local public health alcohol harm strategy	☐	☐	☐	☐
Other	☐	☐	☐	☐

16.a. If you selected other, please specify

17. How often do you discuss specific alcohol licence applications with other Responsible Authorities?

	For every application	Regularly	Occasionally	Never
Police	☐	☐	☐	☐
Licensing team	☐	☐	☐	☐
Fire service	☐	☐	☐	☐
Environmental health / protection (or equivalent)	☐	☐	☐	☐
Children's services	☐	☐	☐	☐
Trading standards	☐	☐	☐	☐
Planning	☐	☐	☐	☐
Other	☐	☐	☐	☐

17.a. If you selected other, please specify

18. Are there formal Responsible Authority meetings in your Local Authority?

18.a. If yes, approximately how often are these meetings?

18.b. If yes, how often are these attended by a public health representative?

19. To your knowledge, have you or your public health colleagues recieved any training on intervening in the alcohol licensing process?

[ ]

19.a. If other, please specify

[ ]

20. How influential do you feel public health is in shaping the alochol licensing work in your Local Authority?

- ☐ Very influential
- ☐ Quite influential
- ☐ Not very influential
- ☐ Not at all influential

20.a. Any additional comments about the alcohol licensing work in your Local Authority:

[ ]

Page 6: Thank you!

Thank you for taking the time to complete this survey.

If you have any questions about this questionnaire or the wider study, please contact Jessica Engen at London School of Hygiene & Tropical Medicine - jessica.engen@lshtm.ac.uk, 020 7927 2380 or Joanna Reynolds - Joanna.reynolds@lshtm.ac.uk, 020 7927 2023.

Key for selection options

1 - By completing this questionnaire, I understand that I give my consent for my responses to be included in the study described above

Yes, I give my consent

15 - How often do you feel you do not have time or capacity to take action on applications within the specified deadline?

Always
Often
Occasionally
Never

18 - Are there formal Responsible Authority meetings in your Local Authority?

Yes
No
Don't know

19 - To your knowledge, have you or your public health colleagues recieved any training on intervening in the alcohol licensing process?

No training
Yes, training by Safe Sociable London Partnership on the SSLP toolkit
Yes, training on the SSLP toolkit by another (eg: colleague)
Yes, other training
