# Supplementary material for: Processes, practices and influence: a mixed methods study of public health contributions to alcohol licensing in local government
Source: BMC Public Health. 2018 Dec 18;18:1385. doi: 10.1186/s12889-018-6306-8 (PMC6299525; doi:10.1186/s12889-018-6306-8)
Supplement: Supplementary file 2 — Sample semi-structured interview topic guide for public health alcohol lead. Sample topic guide used for semi-structured interviews. (PDF 342 kb) [file 12889_2018_6306_MOESM2_ESM.pdf]

Sample semi-structured interview topic guide for public health alcohol lead

This topic guide was modified and adapted for each participant, to reflect their specific role and professional context.

Introduction:

Introduce yourself and the study. Explain that the interview will take around one hour and that they are free to stop the interview or decide not to answer any questions.

Domain	Suggested Questions
**Introductory questions**	Please can you tell me about your role here at _______?<br><br>• *How long have you been in this role?*<br>• *What are the key responsibilities of your role?*
**The role and structure of alcohol licensing work within public health**	Can you tell me about the key strategies relating to alcohol and reducing alcohol-related harms in your public health department?
	What role does alcohol licensing work play alongside your broader work in reducing alcohol-related harms?
	Can you tell me how the alcohol licensing work is structured and conducted within your team?
**Priorities and taking action on applications**	What kinds of priorities shape your (or your team's) decisions around when to take action on alcohol licence applications?
	How often do you submit formal representations on applications?
	What kinds of conditions do you regularly request for licence applications?
	What sources of data or evidence do you typically draw on when screening applications and making representations?
	What influences your decision *not* to take action on a licence application, or to withdraw a representation?

	Can you give me an example of when you have not submitted a representation, or have withdrawn a representation even though you had concerns about an application?
**Relationships with other responsible authorities and the council**	Can you tell me about the relationships between public health and other responsible authorities in relation to the alcohol licensing processes at _______?
	To what extent do these relationships shape how public health make decisions in relation to licence applications?
	What do you think are the priorities of the council and the licensing committee here at _______?
**Success and challenges**	What do you see as the main purpose of public health involvement in alcohol licensing work?
	How successful do you feel public health is in terms of what you seek to achieve through the alcohol licensing work?
	Can you give me an example of when you feel public health has been successful through its alcohol licensing work?
	What do you think are the main challenges you face in terms of doing alcohol licensing work?
	How do you think these challenges limit the kinds of impact you can have through the alcohol licensing work?
	Can you give me an example of when you felt the work was limited or constrained?
	What do you think could help strengthen your alcohol licensing work and its impacts?
	To what extent do you think a fifth licensing objective relating to health would influence your alcohol licensing work and its impacts?
**Engagement with PHAL guidance tool** (*only for areas that have been using the tool*).	(*To PH practitioners only)* I understand you and your colleagues have been using the Public Health Alcohol Licensing guidance tool. Can you tell me about how and why it was introduced?

Again, questions might have to be modified depending on participant's awareness and understanding of the tool.	What have been your experiences of using the tool?
	To what extent do you think the tool supports your alcohol licensing work?
	To what extent do you think the tool has had an impact on your alcohol licensing work and its impacts?
**Non-engagement with PHAL tool** (*for PH practitioners in areas not using the tool)*	What do you know about the Public Health Alcohol Licensing guidance tool developed by Safe Sociable London?
**Summary**	Are there any other points or comments you'd like to make regarding the alcohol licensing work undertaken by public health in ______?
